# Free Energy Profiles Relating With Conformational Transition of the Switch Domains Induced by G12 Mutations in GTP-Bound KRAS

**DOI:** 10.3389/fmolb.2022.912518

**Published:** 2022-05-02

**Authors:** Jianzhong Chen, Shaolong Zhang, Qingkai Zeng, Wei Wang, Qinggang Zhang, Xinguo Liu

**Affiliations:** ^1^ School of Science, Shandong Jiaotong University, Jinan, China; ^2^ School of Physics and Electronics, Shandong Normal University, Jinan, China

**Keywords:** KRAS, G12 mutations, MR-GaMD simulations, free energy profiles, electrostatic environment

## Abstract

Mutations of G12 in KRAS have been involved in different cancers. Multiple replica-Gaussian accelerated molecular dynamics (MR-GaMD) simulations are applied to investigate conformational changes of the switch domains caused by G12C, G12D and G12R. Free energy landscapes suggest that G12C, G12D and G12R induce more energetic states compared to the GTP-bound WT KRAS and make the conformations of the switch domains more disordered, which disturbs bindings of KRAS to effectors. Dynamics analyses based on MR-GaMD trajectory show that G12C, G12D and G12R not only change structural flexibility of the switch domains but also affect their motion behavior, indicating that these three mutations can be used to tune the activity of KRAS. The analyses of interaction networks verify that the instability in interactions of the GTP with the switch SⅠ plays an important role in the high disorder states of the switch domain. This work is expected to provide useful information for deeply understanding the function of KRAS.

## Introduction

In exploration of cancer treatments, RAS proteins have been investigated with intense interest because of its high frequency of mutations in cancers mainly involved in the active site residues G12, G13 and Q61 (Prior et al., 2012; Zeng et al., 2021). RAS proteins share a common feature that cycles between the GTP-bound active state and GDP-bound inactive one. They are functionally used as a molecular switch to regulate vital signaling pathways relating with cell proliferation, differentiation, and apoptosis (Simanshu et al., 2017). GTPase activating proteins (GAPs) promote hydrolysis of GTP into GDP, leading to the GDP-bound inactive form (Scheffzek et al., 1997; Lu et al., 2019), while guanine nucleotide exchange factors (GEFs) trigger release of GDP, resulting in the GTP-bound active state (Bos et al., 2007). Through the cycle, RAS proteins endure large conformational changes and rearrangements. Moreover specific-mutations also induce obvious conformational alterations of RAS proteins and tune the RAS activity. Of the three subfamily members HRAS, KRAS and NRAS, KRAS is the most frequently mutated in cancers (Stephen Andrew et al., 2014; Pylayeva-Gupta et al., 2011). Therefore it is necessary to elucidate influences of specific mutations on conformational dynamics of KRAS for discovering effective direct approaches to addressing RAS-driven cancers (Montalvo et al., 2017; Haigis 2017).

**FIGURE 1:**
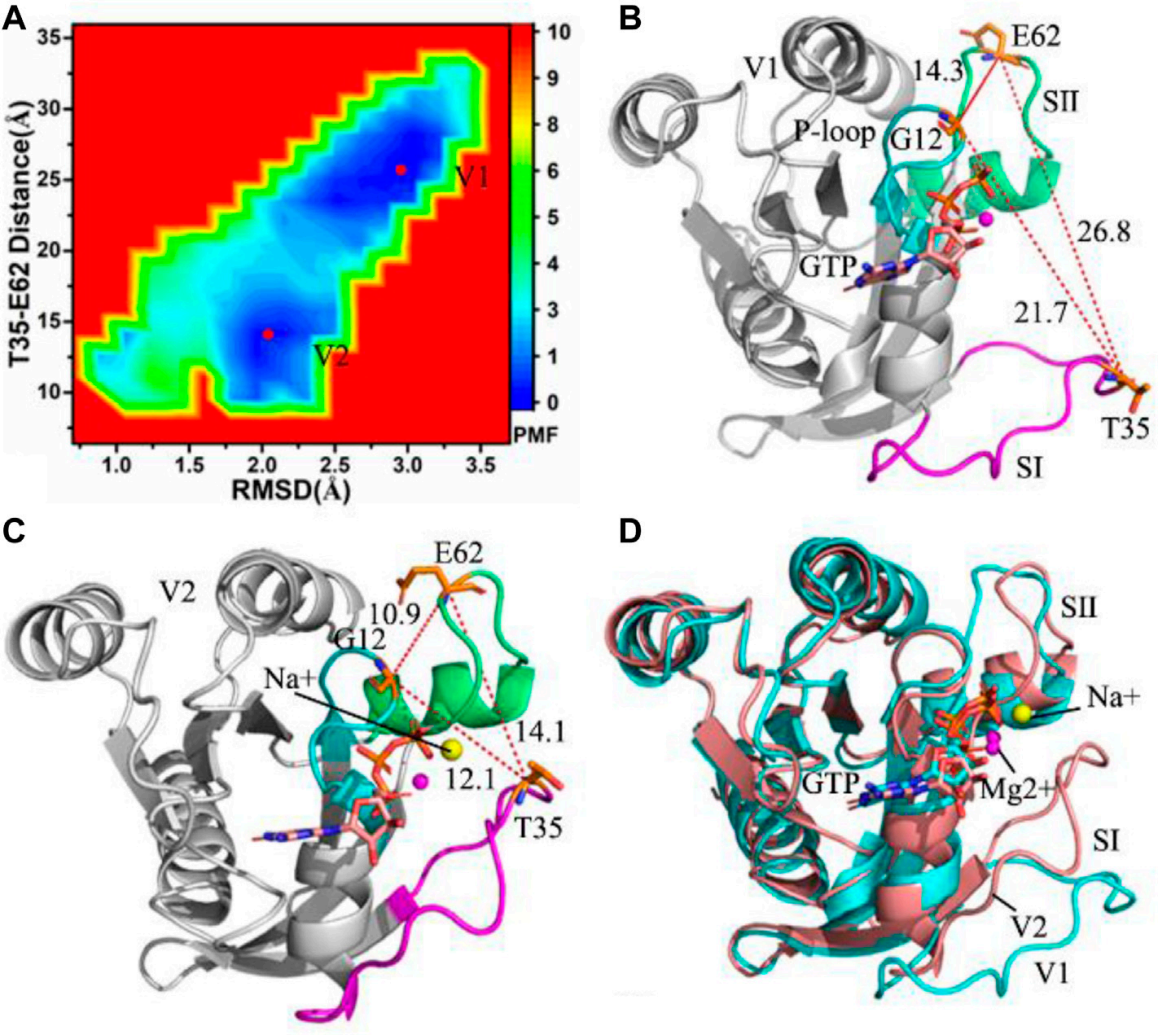
Free energy landscapes and representative structures of the GTP-bound WT KRAS: **(A)** free energy landscape constructed with RMSDs of backbone atoms and the distance between T35 and E62, **(B)** representative structure located at energy valley V1, **(C)** representative structure situated at energy valley V2 and **(D)** superimposition of two representative structures.

The conformational change of KRAS mostly stems from the switch domains of KRAS consisting of the switch Ⅰ (SⅠ, residues 25–40) and switch Ⅱ (SⅡ, residues 59–75). These two switches together with the P-loop (Supporting Information Supplementary Figure S1) encircle the nucleotide-binding pocket and interaction sites of KRAS with its effector and regulator proteins (Parker et al., 2018) (Figure 1A). G12 mutations occurring at the P-loop highly affect conformational rearrangement of the switch domains and efficiently regulate the activity of KRAS (Hunter et al., 2015; Lu et al., 2016). Of G12 mutations, G12C, G12D and G12R are paid more attentions because of differential structural adaptions and fluctuations caused by them. More importantly, G12C, G12D and G12R are involved in the impairment of the KRAS activity and induce malignant tumors (Hobbs et al., 2020; Wang et al., 2022). By comparison, these three residue mutations lead to difference of electrostatic environment around the P-loop. In details, G12C extends the length of hydrophilic sidechain relative to G12 and mutations of G12 into D12 and R12 respectively bring a net negative charge and positive one, which directly produce obvious effect on interactions with GTP that brings a negative charge. Thus, it is of high significance to probe impacts of changes in electrostatic environment on binding of GTP to KRAS and conformational alterations of KRAS for deeply understanding the regulation of the KRAS activity.

Molecular Dynamics (MD) simulations (Xue et al., 2018a; Sun et al., 2021a) and free energy analysis (Sun et al., 2014a; Sun et al., 2014b; Xue et al., 2018b; Sun et al., 2021b) have been extensively applied to decipher binding mechanism of ligands to receptors. To overcome shortcomings of conventional molecular dynamics (cMD) in conformational samplings, Gaussian accelerated molecular dynamics (GaMD) (Miao et al., 2015; Wang et al., 2021) is proposed to improve conformational samplings, moreover this method has been used to successfully explore ligand-receptor binding mechanism (Wang and Miao 2019; Wang and Miao 2020; Chen et al., 2021a; Chen et al., 2021b). In this work, to decode influences of changes in electrostatic environments of the P-loop on the activity of KRAS, multiple replica GaMD (MR-GaMD) simulations are performed on the GTP-bound wild-type (WT), G12C, G12D and G12R KRAS to obtain rational conformational sampling. Principal component analysis (PCA) (Levy et al., 1984), dynamics cross-correlation map (DCCM) calculations (Ichiye and Karplus 1991) and construction of free energy landscapes were carried out to explore G12 mutation-mediated conformation transformation of KRAS. This work is expected to provide useful information for understanding roles of KRAS in anticancer drug design.

## Methods

### System Setup

Due to the lack of the GTP-bound WT KRAS structure, the GDP-bound WT KRAS is taken from protein data bank (PDB) and its entry is 5W22 (Xu et al., 2017). By removing GDP from the superimposed structures of 5W22 with the crystal structure of KRAS complexed with GTP (5VQ2) (Xu et al., 2017), the GTP-bound WT KRAS structure is obtained. To keep atomic coordinate consistence, the GTP-bound G12C, G12D and G12R KRAS are produced through mutations of G12 into C12, D12 and R12 with the Leap module in Amber 20 (Salomon-Ferrer et al., 2013a). A magnesium ion (Mg2+) in the crystal structure is kept at the starting model. The program H++ 3.0 (Anandakrishnan et al., 2012) is adopted to check and assign the protonated state of each residue in KRAS. Meanwhile the Leap module in Amber 20 is utilized to complete the following process of system parameterization: 1) all missing hydrogen atoms in the crystal structure are connected to the corresponding heavy atoms, 2) the parameters of the WT and mutated KRAS are generated by using the *ff*19SB force field (Tian et al., 2020), 3) the parameters of GTP are taken from the work of Meagher et al. (Meagher et al., 2003), 4) an octahedral periodic box of water with a buffer of 12.0 Å is adopted to solve the GTP-bound WT and mutated KRAS and the force field parameters of water molecules are assigned with the TIP3P model (Jorgensen et al., 1983) and 5) the appropriate number of sodium ions (Na+) is added around each complex in salt strength of 0.15 M NaCl to build a neutral simulated system, in which the parameters of Na+, Cl- and Mg2+ are extracted from the Aqvist force field (Ȧqvist 1990).

### Multiple Replica Gaussian Accelerated Molecular Dynamics Simulations

To relieve high-energy contacts between atoms, each system is optimized using the steepest descent minimization of 300 ps and the conjugate gradient one of another 300 ps. Subsequently, the system endures a 1-ns soft heating process from 0 to 300 K by restraining heavy atoms of the GTP-KRAS complex with 1 kcal/(mol·Å^2^) harmonic constant in a constant number, volume and temperature (NVT) ensemble. Then, each system is further equilibrated for 1 ns in a constant number, pressure and temperature (NPT) ensemble at 1 atm and 300 K by using the same restraints as in the NVT simulation. After that, 3-ns cMD simulations are conducted to check potential energy statistics, involving the maximum, minimum, average, and standard deviation of the simulated system. After running the 20-ns GaMD equilibration with the boost potential, three separate 600-ns GaMD simulations are carried out with randomized initial atomic velocities to relax each system. Through the current cMD and GaMD simulations, all chemical bonds linking with hydrogen atoms are restrained with the SHAKE algorithm (Ryckaert et al., 1977). The temperatures of four simulated systems are controlled with the Langevin dynamics with a collision frequency of 2.0 ps^−1^ (Izaguirre et al., 2001). An appropriate cutoff value of 12 Å is used to compute electrostatic interactions with the particle mesh Ewald (PME) method (Essmann et al., 1995), meanwhile this cutoff is also applied to estimate van der Waals interactions. To be convenient for the post processing analysis, three separate replica GaMD trajectories are integrated a single joined trajectory (SJT). The PyReweighting toolkit (Miao et al., 2014) is employed to reweight the data arising the CPPTRAJ analysis on the SJT (Roe and Cheatham 2013) and recover the original free energy of four simulated systems. The details of MR-GaMD simulations, DCCM calculations and PCA have been clarified in our previous works (Chen et al., 2021c). All simulations in this current work are run by aid of the program pmemd. cuda inlayed in Amber 20 (Salomon-Ferrer et al., 2013b).

## Results

### Free Energy Profile of GTP-Bound WT KRAS

To uncover energetic basis of conformational changes, root-mean square deviations (RMSDs) of backbone atoms and the distance of residues T35 away from E62 in the GTP-bound KRAS are used as reaction coordinates (RCs) to build free energy landscapes (FELs), shown in Figure 1A. RMSDs can rationally reflect total structural fluctuations of KRAS. Residue T35 is located at the switch SⅠ and E62 is situated at the switch SⅡ, hence the changes in the distance between T35 and E62 can embody conformational alterations of the switch domains (SⅠ and SⅡ) in KRAS. The aforementioned factors are the reason why we choose them as reaction coordinates.

MR-GaMD simulations capture two energetic valleys (V1 and V2) in the GTP-bound WT KRAS (Figure 1A). In the V1 and V2 states, the distances between T35 and E62 are 26.8 and 14.1 Å (Figures 1B,C), respectively. The distances of G12 away from T35 and E62 in the V2 state are shorter than that in the V1 state (Figures 1B,C). Based on these two facts, the P-loop, SⅠ and SⅡ in the V2 state encircle a more compact switch domain than that in the V1 state. Two representative structures located at the V1 and V2 state are aligned together, which is shown in Figure 1D. The results display that the switch domains (SⅠ and SⅡ) have obvious structural deviation and generate highly structural disorder. It is well known that the SⅠ and SⅡ are involved in binding of KRAS to GEFs and GAPs, hence the conformational disorder of SⅠ and SⅡ certainly affects the activity of KRAS. Despite obvious alterations in the switch domains, the GTP and magnesium ion (Mg2+) are aligned well, only the hydrophobic ring in the middle of GTP produces a slight deviation (Supplementary Figure S2). Meanwhile, a sodium ion (Na+) appears at the V2 structure, which provides an electrostatic compensation for the changes in electrostatic environments caused by conformational alterations.

### Free Energy Profiles of the GTP-Bound G12C, G12D and G12R KRAS

**FIGURE 2:**
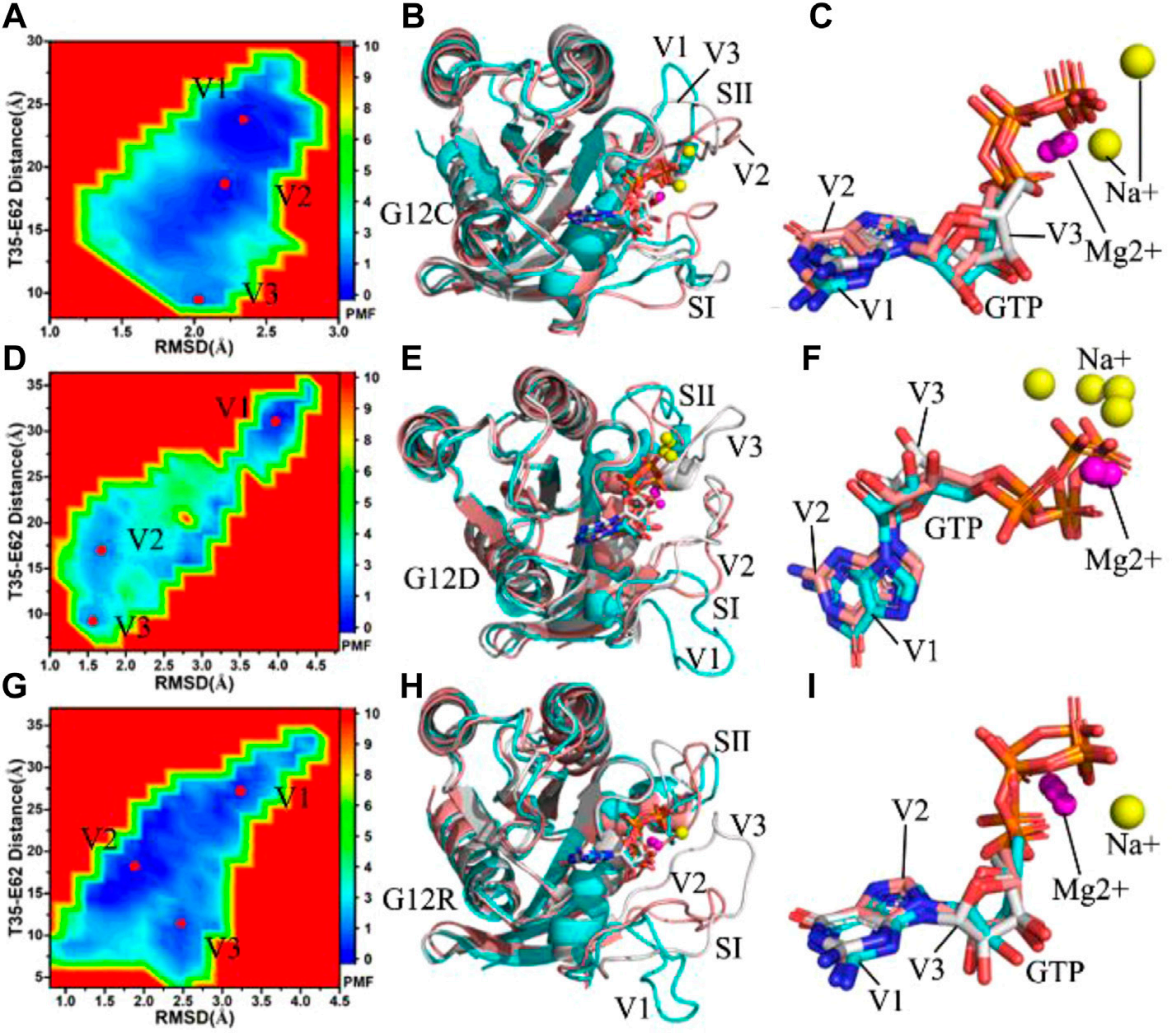
Free energy landscapes and structural information: **(A**,**D,G)** respectively corresponding to free energy landscapes of the GTP-bound G12C, G12D and G12R KRAS, **(B**,**E,H)** separately indicating structural superimpositions of the GTP-bound G12C, G12D and G12R KRAS situated at energetic valleys V1, V2 and V3 and **(C,F,I)** respectively showing structural alignments of GTP and magnesium ions Mg2+ from the GTP-bound G12C, G12D and G12R KRAS located at energetic valleys. KRAS, GTP, magnesium ions Mg2+ and sodium ions Na+ are displayed in cartoon, stick, ball and ball modes, separately.

To reveal impacts of changes in electrostatic environment caused by G12C, G12D and G12R on the conformations of the switch domains, the same reaction coordinates as the WT KRAS are adopted to construct FELs (Figure 2). As a whole, mutations G12C, G12D and G12R at the P-loop yield different influences on free energy profiles of the GTP-associated KRAS, which can provide useful energetic basis for further understanding the function of KRAS.

For the GTP-bound G12C KRAS, three main energetic valleys (V1, V2 and V3) are captured by MR-GaMD simulations (Figure 2A). The distances of T35 away from E62 in the V1, V2 and V3 sates are 23.1, 18.0 and 9.3 Å, respectively, in the meantime, the distances of C12 away from T35 and E62 change obviously (Supplementary Figures S3A–C). Among three representative structures, the switch domains of the V1 structure are the most incompact (Supplementary Figure S3A), while that of the V3 structure are the most compact (Supplementary Figure S3C). Superimposition of three representative structures located at the V1, V2 and V3 states shows that G12C leads to a more disordered conformations of the switch domains by comparison with the GTP-bound WT KRAS (Figure 2B), which changes the position of SⅠ and SⅡ relative to the GEFs and GAPs and affects their bindings. Different from the disordered switches, GTP and magnesium ions (Mg2+) are aligned well in three representative structures apart from the slight sliding of the ring in the middle of GTP (Figure 2C), suggesting the significance of the structural stabilization of GTP and Mg2+ in the function of KRAS. Besides, sodium ions (Na+) appear at the V1 and V3 structures, which compensates the changes of electrostatic environment induced by G12C.

With regard to the GTP-bound G12D KRAS, MR-GaMD simulations scan three energetic valleys V1-V3 (Figure 2D). The distances between T35 and E62 are 31.3, 17.2 and 9.2 Å in the V1, V2 and V3 states (Supplementary Figures S4A–C), respectively. The distances of D12 away from T35 and E62 are highly different from each other among three representative structures. According to Supplementary Figure S4, the most compact switch domain appear at the V3 structure (Supplementary Figure S4C), while the most untight switch domain is observed at the V1 structure (Supplementary Figure S4A). As noted from superimposition of the V1, V2 and V3 structures (Figure 2E), the switch domains generate evident deviations from each other, and the SⅠ in the V1 structure extremely extend outside, which efficiently tunes the activity of KRAS. In spite of so, the structures of GTP and Mg2+ highly agree with each other in three representative structures (Figure 2F). G12D brings a net negative charge for the GTP-bound KRAS and changes the electrostatic environment around the P-loop. To compensate the alterations of electrostatic situations, double, single and single Na + separately appear at the V1, V2 and V3 structures, implying the important role of electrostatic environment in the function of KRAS.

As for the GTP-bound G12R KRAS, three main energetic valleys (V1, V2 and V3) are detected through MR-GaMD simulations (Figure 2G). In the V1, V2 and V3 states, the distances between T35 and E62 are 26.8, 18.4 and 10.8 Å, respectively (Supplementary Figures S5A–C), meanwhile the distances of R12 away from T35 and E62 in the V2 and V3 states are shorter than that in the V1 states. Based on these facts, the switch domains of the V1 structure form the most incompact conformation, while that of the V3 structure produce the tightest topology. Through superimposition of three representative structures, two switch domains (SⅠ and SⅡ) display high disorders, moreover the SⅠ in the V1 structure extremely extends outside and leaves the GTP binding site (Figure 2H). Because the SⅠ and SⅡ are located at the binding regions to the GEFs and GAPs, thus the high disorder of the switch domains certainly generates significant effect on associations of KRAS with effectors. Different from the disorder of the SⅠ and SⅡ, the GTP and Mg2+ are aligned well in three states (Figure 2I), suggesting that GTP and Mg2+ are stable through MR-GaMD simulations and play an important role in the function of KRAS. Besides, a sodium (Na+) is detected at the V3 structure (Supplementary Figure S5C), which provides a compensation for the changes of electrostatic environment in the compact state caused by G12R.

Based on the aforementioned analyses, compared to the most incompact switch domains of the GTP-bound WT KRAS, the distances of T35 away from E62 is increased by 4.5 Å due to G12D and reduced by 3.7 Å because of G12C. This result indicates the changes in electrostatic environments caused by G12 mutations generate different effect on the conformation of the switch domains, which should be paid more attentions in understanding the function of KRAS.

### Changes in Conformational Dynamics of KRAS Induced by G12C, G12D and G12R

To understand effect of G12C, G12D and G12R on conformational dynamics of KRAS, difference in root-mean-square fluctuations (RMSFs) of the C_α_ atoms is estimated with the SJT (Supplementary Figure S6A). G12C, G12D and G12R yield strong impacts on the conformations of the switch domains. G12D and G12R strengthen structural flexibility of the SⅠ, while G12C weakens that of this switch. Differently, G12C and G12D highly reduce the structural flexibility of the SⅡ, but G12R obviously enhances that of the SⅡ. Molecular surface areas (MSAs) of the WT and mutated KRAS are also calculated by using the SJT (Supplementary Figure S6B). By comparison with the WT KRAS, G12C and G12D respectively lead to the decrease of 144.9 and 141.1 Å^2^ in MSAs of KRAS, while G12R results in an increase of 141.1 Å^2^, implying that G12C and G12D weaken the total flexibility of KRAS but G12R strengthens that of KRAS.

DCCMs are computed by using the coordinates of the C_α_ atoms saved at the SJT (Supplementary Figure S7) to check the internal dynamics of KRAS. In the WT KRAS, the switches SⅠ and SⅡ produce strong anticorrelated motions (blue and dark blue) relative to the P-loop, meanwhile the SⅡ also generates obvious anticorrelated movements relative to the SⅠ (Supplementary Figure S7A). By referencing the WT KRAS, G12C obviously strengthen the anticorrelated motion between the SⅠ and the P-loop (Supplementary Figure S7B), but G12D and G12R slightly weaken this anticorrelated motion (Supplementary Figures S7C,D). Compared to the WT KRAS, G12D leads a complete disappearance of the anticorrelated motions between the SⅡ and the P-loop (Supplementary Figure S7C), on the contrary G12C and G12R softly enhance this anticorrelated motion (Supplementary Figures S7B,D). As a common phenomenon, all of three mutations increase the anticorrelated motion between the SⅡ and SⅠ compared to the WT KRAS (Supplementary Figures S7B–D).

To capture the concerted motions of the structural domains, the first eigenvector from PCA is used to characterize the domain motions of KRAS (Supplementary Figure S8). It is found that the switch domains SⅠ and SⅡ exhibit not only high concerted motion but also the strong motion behavior in the WT and mutated KRAS, further verifying that the SⅠ and SⅡ are extremely flexible. In the WT KRAS, the SⅠ and SⅡ move in an opposite direction and are close to each other, furthermore the part of the SⅠ (SⅠ-L1) moves outside (Supplementary Figure S8A). Compared to the WT KRAS, although G12C and G12R hardly change the motion strength of the SⅠ and SⅡ, they evidently alter the motion direction of the SⅠ-L1 (Supplementary Figures S8B,D). Interestingly, G12D not only completely alters the motion tendency of the SⅠ and SⅡ but also inhibits the motion strength of the switch domains relative to the WT KTAS (Supplementary Figure S8C).

### Alterations in Interaction Network of GTP With KRAS Caused by G12C, G13D and G12R

**FIGURE 3:**
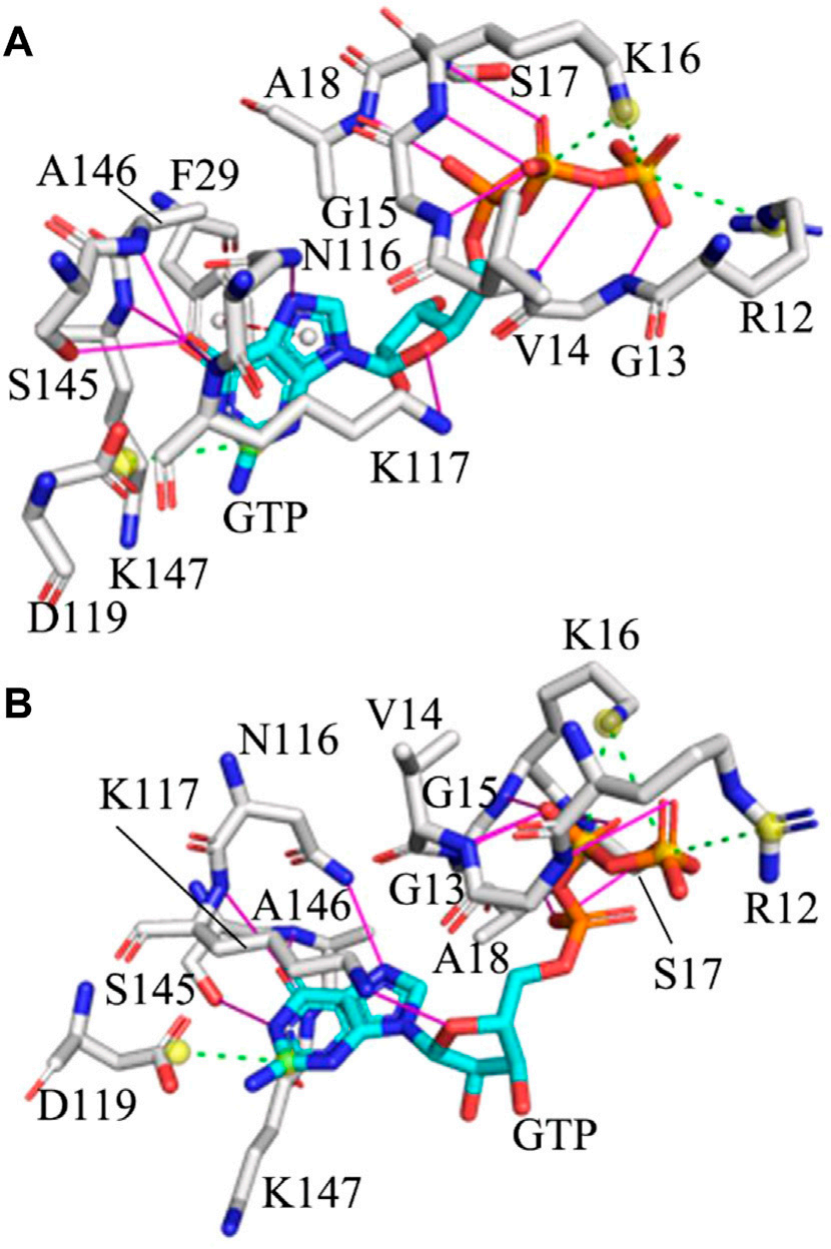
Interaction network of GTP with the G12R KRAS: **(A)** and **(B)** respectively corresponding to interactions of GTP with key residues in the situation of the most incompact and tightest switch domains. Hydrogen bonding interactions, salt bridge and π-π interaction are indicated in the magenta full line, green dot line and red dot line, separately.

Interaction networks of GTP with KRAS are analyzed by using a protein-ligand interaction profiler (PLIP) server (Salentin et al., 2015) and the results are provided at Figure 3 and Supplementary Figures S9–S11. It is observed that hydrogen bonding interactions (HBIs), salt bridge and π-π interactions are the main force of the GTP-KRAS binding. In this work, only the structures located at the tightest and most incompact switch domains are adopted to analyze interaction networks of GTP with the KRAS.

Based on comparison between the tightest and most incompact situations of the WT, G12C, G12D and G12R KRAS, it is found that HBIs of GTP with G13, V14, G15, S17, A18, N116, K117, A146 and K147 exist at the WT and mutated KRAS, showing that these HBIs are stable and they play an important role in keeping the GTP-KRAS binding (Supplementary Figures S9–S11 and Figure 3). In the meantime, three salt bridge interactions between GTP and KRAS are observed at all studied systems, in which the carbonyl group of D119 forms a salt bridge with the guanine group of GTP while the positive charge group of K16 produces two salt bridges with the PB and PG groups in the phosphate of GTP (Supplementary Figures S9–S11 and Figure 3). A HBI of GTP with D30 appears at the situations of the tightest switch domain from the WT, G12C and G12D KRAS, but this HBI disappears at the most incompact state. Thus the instability of the HBI between GTP and D30 drives the conformation disorder of the SⅠ. In addition, G12C and G12D induce a disappearance of a HBI between A11 and GTP in the tightest situation of the G12C and G12D KRAS compared to that of the WT KRAS (Supplementary Figure S9B, Supplementary Figure S10B and Supplementary Figure S11B). Different from the WT, G12C and G12D KRAS, G12R induces the appearance of a π-π interaction between F29 of the SⅠ and the guanine group of GTP at the situation of the most incompact switch domain from the G12R KRAS, which shows that the interaction of GTP with the SⅠ is instable during MR-GaMD simulations.

## Discussion

Based on FELs, G12C, G12D and G12R induce more energetic states and make the switch domains more disordered compared to the WT KRAS. The switch domains are involved in interactions with GAPs and GEFs, hence more disordered states of the switch domains caused by G12C, G12D and G12R directly affect binding of KRAS to effectors. Although G12C, G12D and G12R generate evident influences on the conformations of the switch domains, they hardly disturb the stability of GTP and magnesium ion (Mg2+) through the entire MR-GaMD simulations, which is supported by the distance distributions between Mg2+ and the phosphate group of GTP (Supplementary Figure S12). It is concluded that the high stability of GTP and Mg2+ are essential for the function of KRAS. G12C, G12D and G12R change the electrostatic environment around the P-loop, sodium ions appear at a certain conformational states and compensate the different electrostatic effects. This study verifies that the G12D mutated KRAS need more sodium, moreover the previous works also demonstrate the electrostatic compensation of sodium ions on the environment changes (Chen et al., 2022). Analysis of RMSFs and MSAs suggest that G12C, G12D and G12R change not only local structural flexibility of the switch domains but also total structural flexibility of KRAS. The results from DCCM calculations indicate that G12C, G12D and G12R affect motion modes of the SⅠ and SⅡ relative to the P-loop and SⅠ. The visualization of the first eigenvector representing mainly concerted motions of KRAS not only verify that the switch domains possess highly strong motions but also suggest that G12C, G12D and G12R produce significant influences on the motion behavior. It is well known that the switch domains are involved in binding of KRAS to GEFs and GAPs, hence the alterations in structural flexibility and conformational dynamics certainly affect associations of KRAS with effectors and tune the activity of KRAS. The analyses of interaction networks further verify that the instability of the GTP-SⅠ interaction plays an important role in the disorder of the switch domains. This work is expected to provide energetic basis and dynamics information for deeply understanding the function of KRAS.

## Data Availability

The original contributions presented in the study are included in the article/Supplementary Material, further inquiries can be directed to the corresponding authors.
