# Supplementary material for: Free Energy Profiles Relating With Conformational Transition of the Switch Domains Induced by G12 Mutations in GTP-Bound KRAS
Source: Front Mol Biosci. 2022 May 2;9:912518. doi: 10.3389/fmolb.2022.912518 (PMC9108337; doi:10.3389/fmolb.2022.912518)
Supplement: Supplementary file 1 [file DataSheet1.PDF]

Free Energy Profiles Relating with Conformational Transition of the Switch Induced by G12 Mutations in GTP-bound KRAS

Jianzhong Chen[1,*], Shaolong Zhang[2], Qingkai Zeng[1], Wei Wang[1], Qinggang Zhang[2] and Xinguo Liu[2,*]

[1] *School of Science, Shandong Jiaotong University, Jinan 250357, China,* [2] *School of Physics and Electronics, Shandong Normal University, Jinan 250358, China*

Correspondence:

Jianzhong Chen: chenjianzhong1970@163.com, jzchen@sdjtu.edu.cn

Xinguo Liu: liuxinguo@sdnu.edu.cn

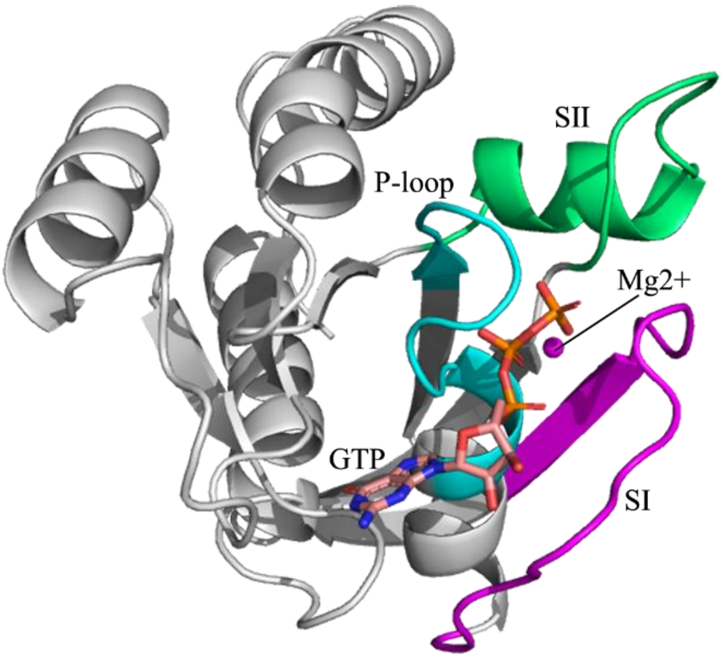

Figure S1. Molecular structure of the GTP-bound WT KRAS: P-loop, switches SI and SII are indicated in cyan, magenta and limegreen, respectively, and KRAS, GTP and magnesium ion (MG2+) are shown in cartoon, stick and ball modes.

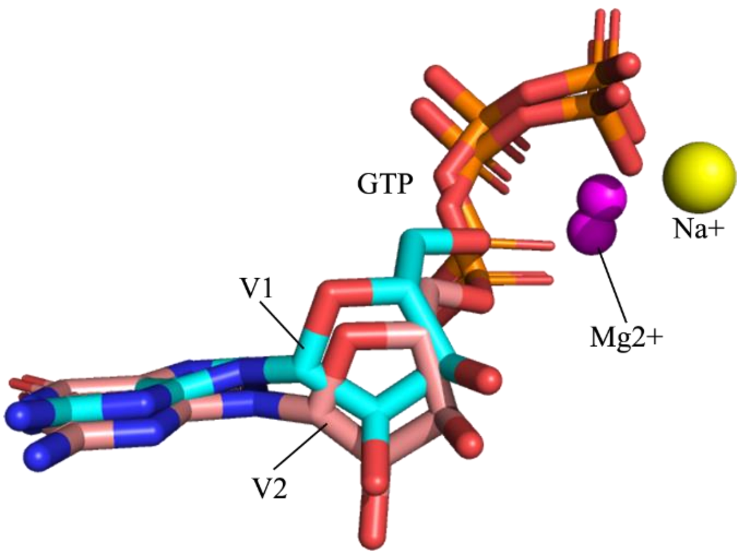

FIGURE S2 | Structural superimposition of GTP and magnesium ions (Mg2+) in two representative structures of the GTP-bound WT KRAS situated at the V1 and V2 states.

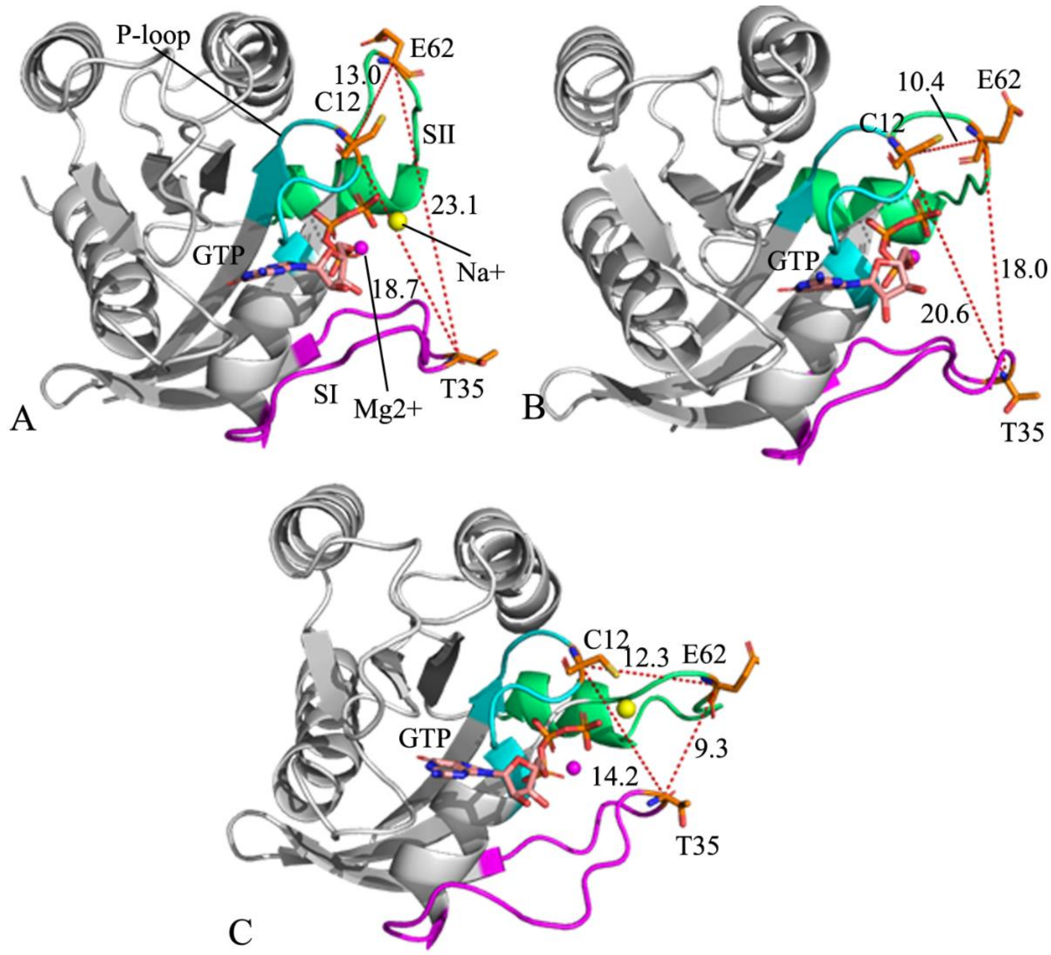

FIGURE S3 | Three representative structures of the GTP-bound G12C KRAS situated at the V1, V2 and V3 sates: (A), (B) and (C) corresponding to the V1, V2 and V3 states, respectively. In this figure, KRAS, GTP, key residues and ions (Mg2+ and Na+) are displayed in cartoon, stick, stick and ball modes, separately.

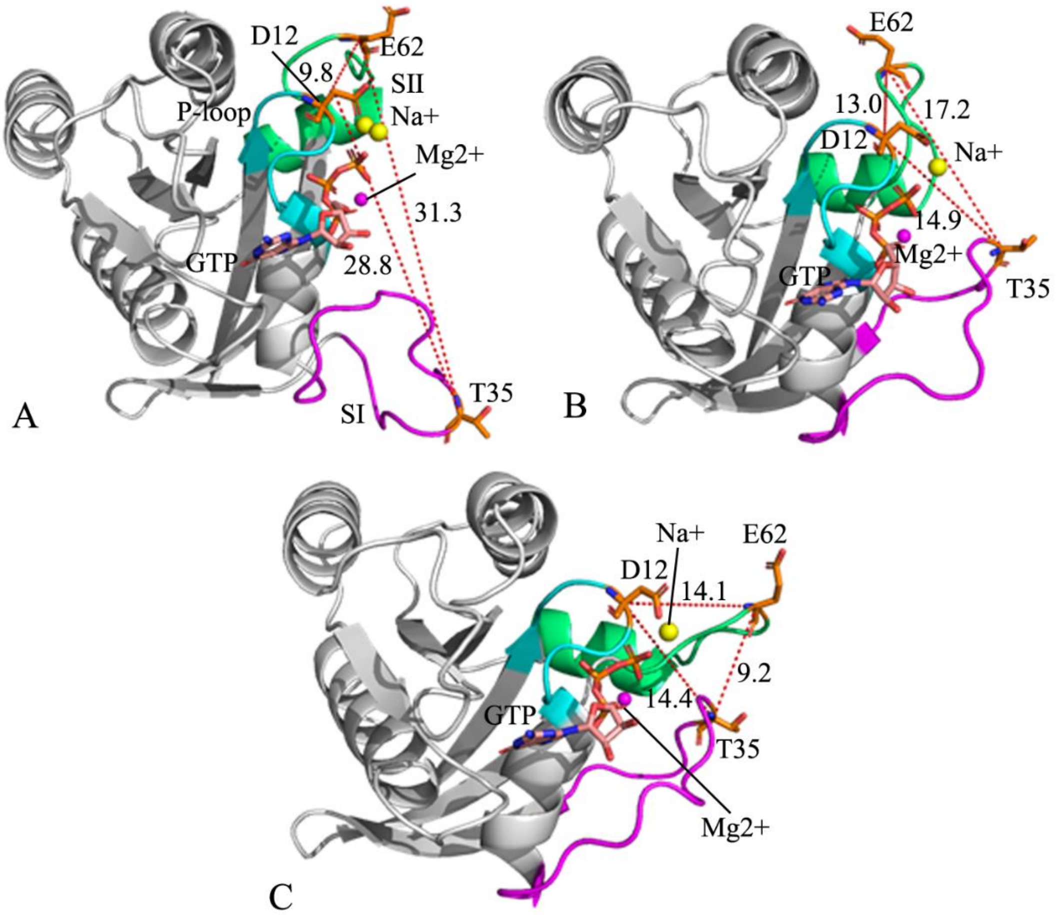

FIGURE S4 | Three representative structures of the GTP-bound G12D KRAS located at the V1, V2 and V3 sates: (A), (B) and (C) representing the V1, V2 and V3 states, independently. In this figure, KRAS, GTP, key residues and ions (Mg2+ and Na+) are shown in cartoon, stick, stick and ball styles, respectively.

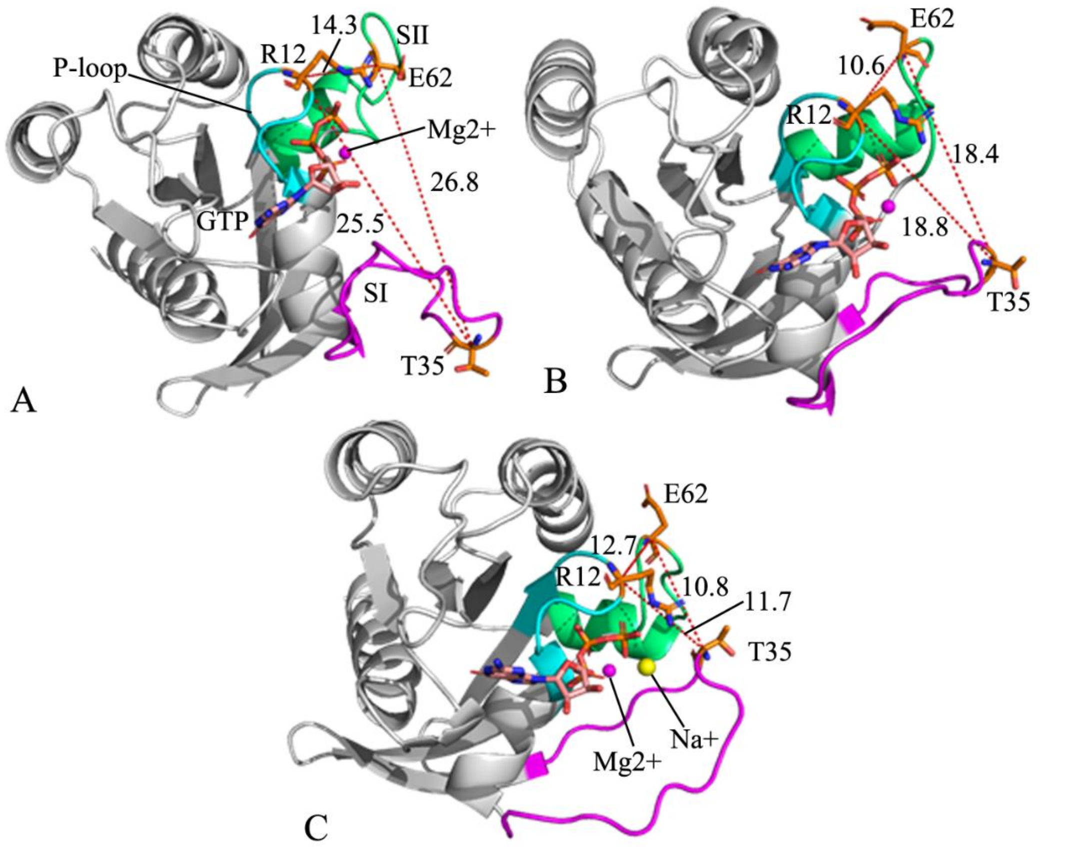

FIGURE S5 | Three representative structures of the GTP-bound G12R KRAS situated at the V1, V2 and V3 sates: (A), (B) and (C) corresponding to the V1, V2 and V3 states, respectively. In this figure, KRAS, GTP, key residues and ions (Mg2+ and Na+) are displayed in cartoon, stick, stick and ball modes, separately.

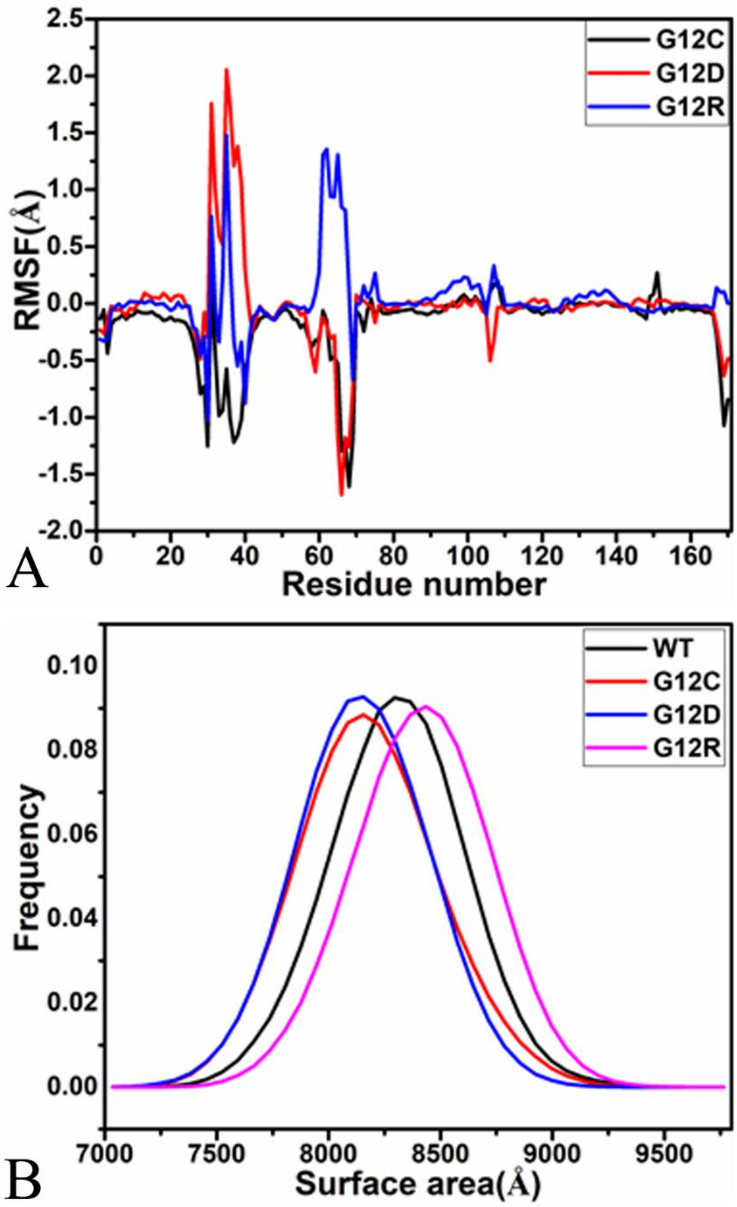

FIGURE S6 | (A) Difference in root-mean-square fluctuations (RMSFs) of C_α atoms from KRAS and (B) molecular surface areas of the GTP-bound WT and mutated KRAS.

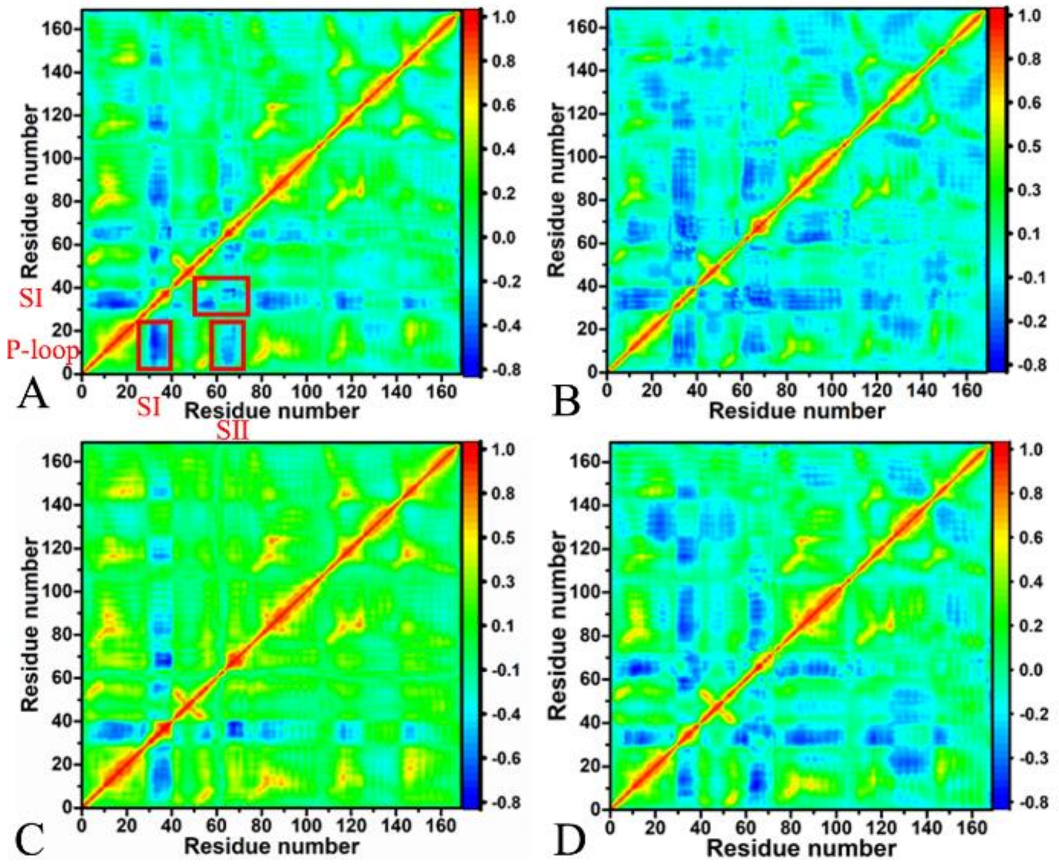

FIGURE S7 | Dynamics cross-correlation map calculated by using the coordinates of Cα atoms recorded at MR-GaMD trajectory: (A) the GTP-bound WT KRAS, (B) the GTP-bound G12C KRAS, (C) the GTP-bound G12D KRAS and (D) the GTP-bound G12R KRAS.

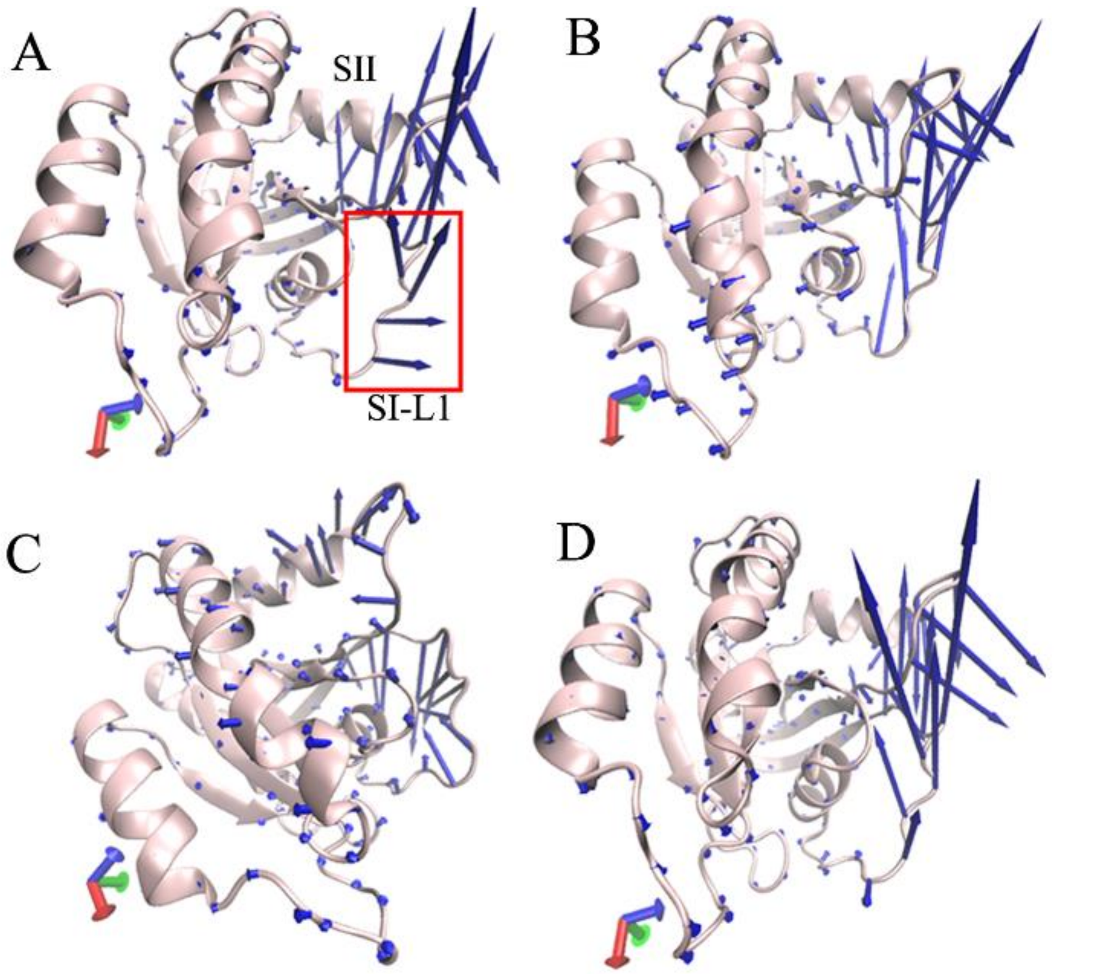

FIGURE S8 | Concerted motions of the structural domains in KRAS captured by the first eigenvector arising from principal component analysis: (A) the GTP-bound WT KRAS, (B) the GTP-bound G12C KRAS, (C) the GTP-bound G12D KRAS and (D) the GTP-bound G12R KRAS.

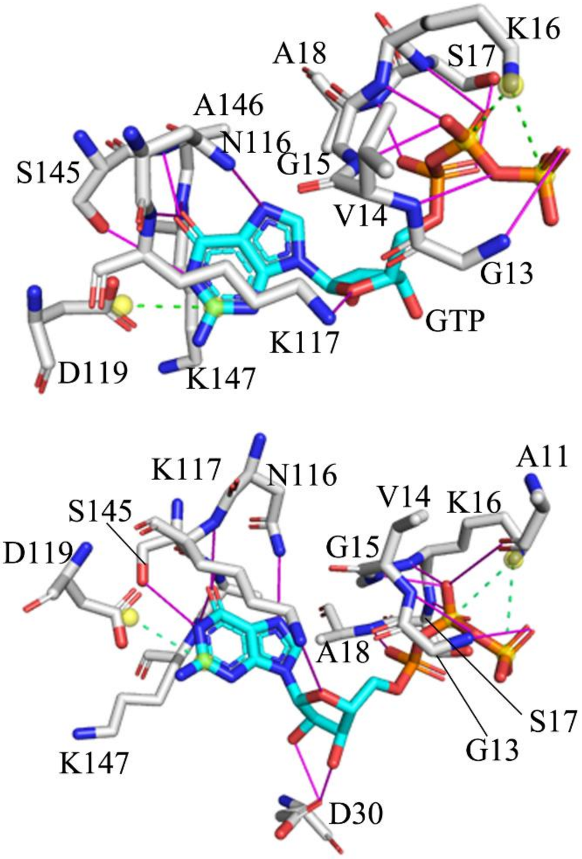

FIGURE S9 | Interaction network of GTP with the WT KRAS: (A) and (B) respectively corresponding to interactions of GTP with key residues in the situation of the most incompact and tightest switch domains. Hydrogen bonding interactions, salt bridge and π-π interaction are indicated in the magenta full line, green dot line and red dot line, separately.

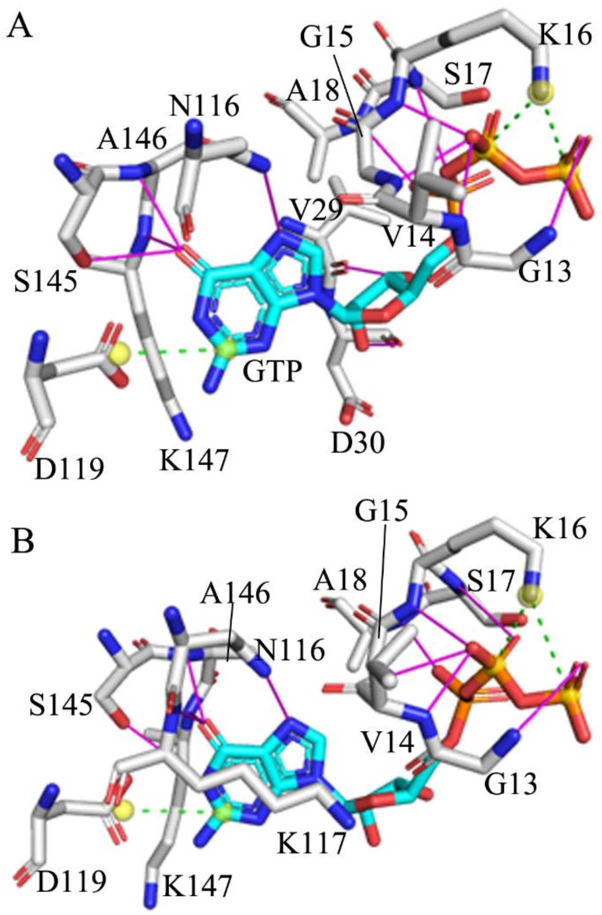

FIGURE S10 | Interaction network of GTP with the G12C KRAS: (A) and (B) respectively corresponding to interactions of GTP with key residues in the situation of the most incompact and tightest switch domains. Hydrogen bonding interactions, salt bridge and π-π interaction are indicated in the magenta full line, green dot line and red dot line, separately.

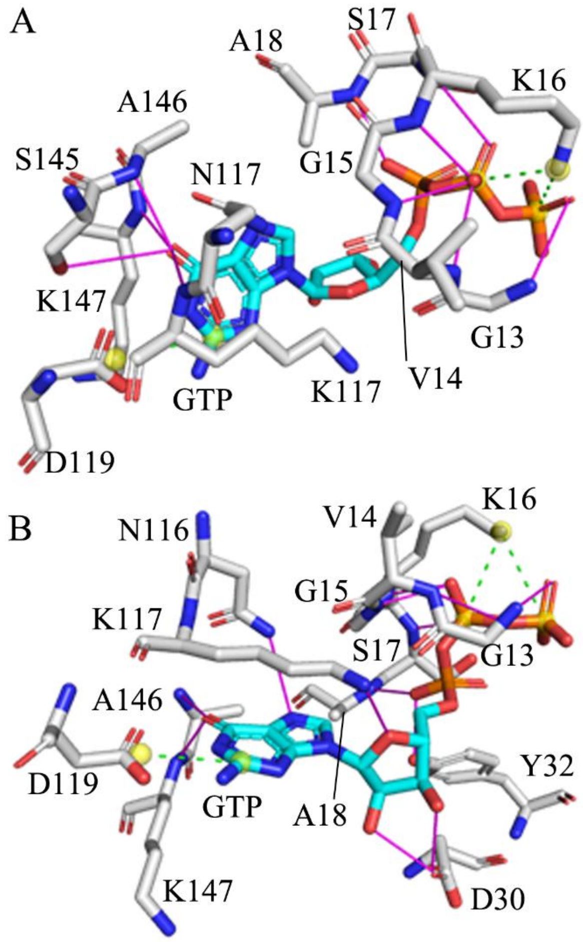

FIGURE S11 | Interaction network of GTP with the G12D KRAS: (A) and (B) respectively corresponding to interactions of GTP with key residues in the situation of the most incompact and tightest switch domains. Hydrogen bonding interactions, salt bridge and π-π interaction are indicated in the magenta full line, green dot line and red dot line, separately.

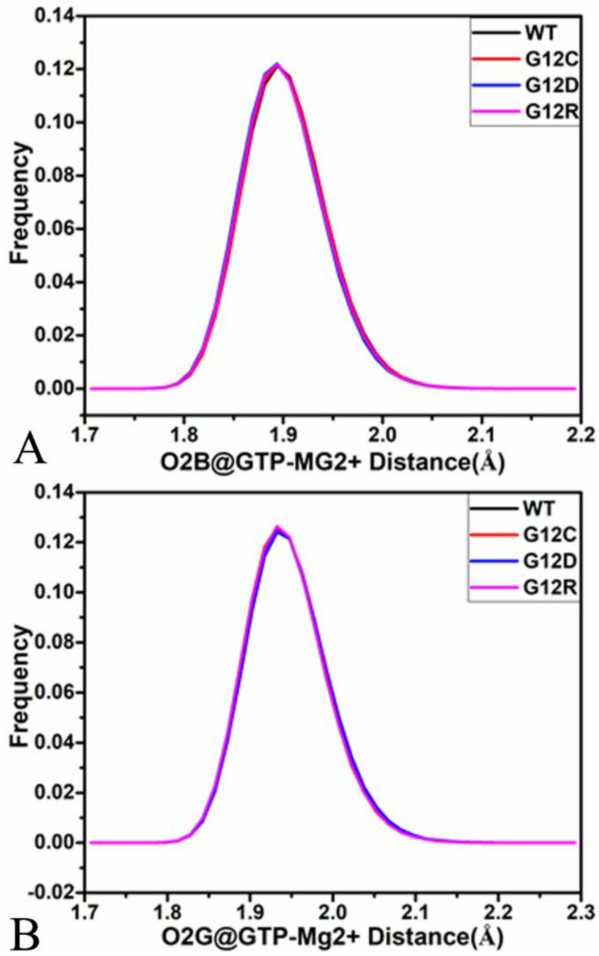

FIGURE S12 | Interactions of magnesium ion (Mg2+) with GTP: (A) interaction betweenMg2+ and oxygen atom O2B of GTP and (B) interaction of Mg2+ with oxygen atom O2G of GTP.
